# Effect of Supplementing Vitamin E, Selenium, Copper, Zinc, and Manganese during the Transition Period on Dairy Cow Reproductive Performance and Immune Function

**DOI:** 10.3390/vetsci10030225

**Published:** 2023-03-15

**Authors:** Yi-Hsuan Chen, Yi-Ming Chen, Po-An Tu, Kuo-Hua Lee, Jih-Yi Chen, Jih-Tay Hsu

**Affiliations:** 1 Taipei Zoo, No. 30, Sec. 2, Xinguang Road., Wenshan Dist., Taipei 116, Taiwan; 2 Department of Animal Science and Technology, National Taiwan University, Taipei 106, Taiwan; 3 Miaoli Animal Care and Health Office, No. 382-1, Yuqing Road., Miaoli City 360, Taiwan; 4 Hsinchu Branch, Livestock Research Institute, Council of Agriculture, Taichung City 368, Taiwan

**Keywords:** transition dairy cow, reproductive performance, immune function, vitamin E, trace elements

## Abstract

**Simple Summary:**

Global demand for dairy products keeps increasing. Yet, dairy cows are exposed to numerous stresses, such as metabolic stress and heat stress, particularly during the transition period, which is three weeks around calving. In the face of climate change, techniques to improve transition cattle both production and reproduction in high temperature and humidity environments are important, particularly in subtropical regions. The present work examined whether supplementation of selenium and vitamin E or zinc, copper, and manganese complex would help transition dairy cows to improve their health and achieve greater milk production by overcoming immune and metabolic stress. The supplementation of trace elements and vitamin E may enable transition cattle to better adapt to heat stress. The conclusions of this study can provide dairy farmers in Taiwan or other subtropical regions with a reference to adjust their management practice for transition dairy cows.

**Abstract:**

The transition dairy cows are challenged by various stresses such as decreased dry matter intake, liver dysfunction, increased inflammation, and oxidative stress, particularly in subtropical regions. These might increase the requirement for vitamin E and trace elements. To examine whether supplementation of vitamin E, selenium or copper, zinc, and manganese complex would help transition dairy cows to achieve greater reproduction performance by overcoming the immune function and postpartum disorders in subtropical Taiwan. A total of 24 Holstein Friesian dairy cows were enrolled in this study and divided into three groups (*n* = 8 cows/group): treatment 1 supplemented with organic selenium and vitamin E (SeE), treatment 2 supplemented with organic copper, zinc, and manganese complex (CZM) and control (CON). The results showed SeE supplementation improved immune function, reproductive performance, and milk yield, but not negative energy balance status. Supplementation of CZM improved milk yield and energy regulation through antioxidative capacity and immune function, but had no influence on reproductive performance.

## 1. Introduction

A transition period in dairy cows is full of stress caused by physiological, metabolic, and nutritional changes [1]. These stresses have an influence on lactation performance, subclinical and clinical postpartum illness, such as ketosis and mastitis, and reproduction disorders, such as retained fetal membranes (RFM), thus significantly affecting profitability [2]. Heat stress in late gestation negatively affects both the dam and offspring [3]. When improving the fertility of dairy cows, we should first strive to maintain immune function, especially when they are exposed to metabolic and physiological stress [4]. Recently, heat stress in late gestation was an economic burden on the US dairy industry [5]. A similar situation exists in Taiwan.

The transition period is characterized by decreased dry matter intake, liver dysfunction, and increased inflammation and oxidative stress [6], which might increase the need for trace elements and vitamin E. Trace elements are important for many critical biological functions including immunity, oxidative metabolism, nutrient, and energy metabolism, and reproductive function in dairy cows [7]. Selenium (Se) plays a role in the antioxidant system as an important part of the glutathione peroxidase enzymes which can remove hydrogen peroxide (H_2_O_2_) and lipid hydroperoxides [8]. Vitamin E has a positive immune function in animals [9]. Copper (Cu) and zinc (Zn) are essential components of several enzymes including Cu-Zn superoxide dismutase (SOD) and the oxidation–reduction process [10]. Manganese (Mn) is a critical co-factor in enzymatic reactions related to metabolic regulation [11]. Furthermore, substantial evidence exists that trace minerals such as Se, Zn, Cu, and Mn can minimize the negative effects of oxidative stress [7]. Cattle in subtropical and tropical regions are subjected to many stress factors, including seasonally poor nutrition, parasites and blood-sucking insects, high temperatures, and high humidity. Heat stress can worsen the existing stress condition of transition dairy cows in subtropical regions. In the face of climate change, techniques to improve both milk production and reproductive performance in high-temperature and humid environments are important. The objective of the present study was to examine whether supplementation of Se and vitamin E (SeE) or Cu, Zn, and Mn complex (CZM) would help transition dairy cows in subtropical regions to achieve greater reproduction performance by overcoming the immune and postpartum disorders.

## 2. Materials and Methods

### 2.1. Cattle and Herd Management

This study was performed in accordance with Taiwan regulations and approved by the Institutional Animal Care and Use Committee of Livestock Research Institute (Approval number: 10714). Pregnant heifers and dry cows were housed together in a free stall. Before calving, each pregnant heifer or dry cow was given 3 kg/day concentrate (Table 1), and free access to pangola grass hay and clean water. After calving, all cattle were fed a total mixed ration (TMR) (Table 2) twice daily (0500 h and 1400 h) and had free access to clean water. The TMR was formulated according to the National Research Council Committee on Animal Nutrition [12]. Main ingredients of TMR were bermudagrass hay, alfalfa hay, corn silage, soybean hulls, brewers’ grains, and concentrates based on corn and soybean meal. Cattle were milked twice daily.

### 2.2. Experimental Design

This study was conducted from April to November 2018 in dairy barn of Hsinchu Branch, Livestock Research Institute in Miaoli County, Taiwan. Using the expected negative energy balance prevalence rate of 80% during early lactation including transition period [13] as a reference, the formula from Thrusfield [14] with a 95% confidence interval and an absolute precision of 5% was used to calculate the proper sample size of the study:

$$n=\frac{1.96^{2}P_{exp}\left(1-P_{exp}\right)/\varepsilon^{2}}{\frac{N-1}{N}+\frac{1.96^{2}P_{exp}\left(1-P_{exp}\right)}{N\varepsilon^{2}}}$$

where n = required sample size; $P_{exp}$ = expected prevalence; N = small finite population size; $\varepsilon^{2}$ = maximum error. Total of 30 Holstein cows with about 180 records was initially planned for the study. During the experiment, 6 cows had to be removed from the experiment due to premature delivery or lameness. In the end, a total of 24 Holstein Friesian cows (11 primiparous cows and 13 multiparous cows) completed the study. All these 24 cows were divided into three groups, treatment 1 group supplemented with 1500 IU vitamin E (E-50 Adsorbate, ROVIMIX^®^, DSM, Heerlen, The Netherlands) and 0.3 ppm of organic Se (zinc-L-selenomethionine, Availa^®^Se, ZINPRO, Eden Prairie, MN, USA) (SeE group, *n* = 8, body condition score (BCS) at the beginning = 3.09 ± 0.27, parity = 1.75 ± 0.71), treatment 2 group supplemented with 12.5 ppm of organic Zn, 5 ppm of organic Cu and 7.5 ppm Mn (amino acid complex, Availa^®^4, ZINPRO, Eden Prairie, MN, USA) (CZM group, *n* = 8, BCS at the beginning = 3.06 ± 0.26, parity = 1.75 ± 0.89) and control group with no extra supplementation (CON group, *n* = 8, BCS at the beginning = 3.06 ± 0.22, parity = 1.75 ± 0.89) from −21 to 21 days of calving (where day 0 is the anticipated calving date). The extra supplementation was first weighed and then mixed with the concentrate to feed the cattle. All cows delivered single calves and were without dystocia or abortions. In this experiment, all cows were under group feeding, so no individual dry matter intake could be obtained. The dry matter intake during last weeks of gestation and early lactation was calculated to be 11.7 kg and 14.5 kg, respectively, plus 10% of the leftover feed.

### 2.3. Monitoring Temperature–Humidity Index of Barn

Temperature and relative humidity in barn were measured using PC sensor water-proof HS10 (Unicom Integration System, Taiwan) every 15 min during experiment period. The temperature–humidity index was calculated based on following equation: temperature–humidity index (THI) = (1.8 × T + 32) − [(0.55 − 0.0055 × RH) × (1.8 × T − 26)], where T = air temperature (°C) and RH = relative humidity (%) [15].

### 2.4. Blood Sampling and Hematologic and Biochemical Profile Examination

Blood samples were analyzed for hematologic and biochemical profiles to evaluate immune function, postpartum health, and disorders. Blood was sampled at d −21, −14, −7 of expected calving, and at d 7, 14, 21 post-calving by jugular venipuncture aseptically using 20 G needle in sterilized vacutainers (EDTA, heparinized or clot activators). EDTA vacutainers were used for blood samples intended for analyzing hematologic complete blood count, β- hydroxybutyrate (BHBA), and glucose, while clot activator vacutainers were used for serum non-esterified fatty acids (NEFA), and heparinized vacutainers were used for plasma biochemical profile analyzing. Serum and plasma were separated by centrifugation at 1500× *g* for 15 min and stored at −20 °C until analysis.

Hematologic complete blood count included white blood cell count (WBC), red blood cell count (RBC), neutrophils, lymphocytes, monocytes, eosinophils and basophils, hemoglobin (HGB), hematocrit (HCT), mean corpuscular volume (MCV), mean corpuscular hemoglobin (MCH), mean corpuscular hemoglobin concentration (MCHC), immediately after sampling, using Hematology Analyzer (D × H 500; Beckman Coulter, Brea, CA, USA). Blood samples were analyzed for BHBA and glucose by test kit (Optium β Ketone Test Strips and Optium Blood Glucose Test Strips; FreeStyle, Abbot, Chicago, IL, USA) immediately after sampling. Serum sample for NEFA using Hitachi 704 Analyzer (Hitachi, Tokyo, Japan) and plasma biochemical sample for aspartate transaminase (GOT), alanine aminotransferase (GPT), γ-glutamyltransferase (GGT), lactate dehydrogenase (LDH), albumin, globulin, total protein, blood urea nitrogen (BUN), creatinine, triglyceride, creatine phosphokinase (CPK), Ca, P, Mg, Na, K, and Cl using 7170 Chemistry Analyzer (Hitachi, Tokyo, Japan).

### 2.5. Postpartum Health or Disorders

From calving to 21 days in milk (DIM), cattle were observed with the incidence of RFM, ketosis, and clinical and subclinical hypocalcemia. For documentation consistency, farm owners recorded postpartum illness on a standard form provided by the research team and each definition of a disease event was discussed with veterinarians. RFM was defined as fetal membranes not being eliminated within 24 h of calving [16]. Subclinical hypocalcemia was characterized as a postpartum circulating concentration of Ca ≤ 2.1 mmol/L (8.42 mg/dL) [17]. Clinical ketosis was defined as elevated blood ketones with depressed appetite and lethargy (postpartum circulating concentration of BHBA ≥ 1.2 mmol/L). Cattle without signs of illness, but with postpartum circulating concentration of BHBA ≥ 1.2 mmol/L was defined as subclinical ketosis [18]. Normal NEFA levels for cows in positive energy balance are less than 0.2 mM [19].

### 2.6. Body Condition Score (BCS)

The BCS was assessed by the same well-trained personnel at −21, −14, −7 prepartum and at 7, 14, and 21 d postpartum using a 5-point scale (1 = thin and 5 = fat) [20].

### 2.7. Milk Production and Composition Recording

Milk yield was recorded at each milking during the whole experiment period by AFIMEN management system (Afimilk Ltd., Kibbutz Afikim, Israel). Weekly milk composition, including milk protein percentage (MPP), milk fat percentage (MFP), milk lactose percentage (MLP), milk solids-not-fat percentage (MSNFP), milk total solid percentage (MTSP), somatic cell counts (SCC), milk urea nitrogen (MUN) and milk citrate (MC) was analyzed and recorded by DHI (dairy herd improvement) laboratory using FOSS MikoScan FT and Fossomatic FC (FOSS, Denmark). The SCC was transformed into somatic cell linear scores (SCLS; ranging from 0 to 9) to obtain data normality for the statistical analysis by the equation: SCLS = [log2 (SCC/100)] + 3, [21].

### 2.8. Measurement of Reproductive Performance

Heat detection was completed by activity monitoring at 15 min intervals daily by Afimilk heat detection system (Afimilk Ltd., Israel). Following the guides for artificial insemination steps (https://www.fwi.co.uk/livestock/livestock-breeding/8-step-guide-artificially-inseminating-dairy-cow, accessed on 15 March 2018), lactating cows were artificially inseminated after heat detection. All animals with non-return of estrus cycle were subjected to pregnancy check by experienced veterinarian using transrectal palpation at 60–65 days post insemination. Postpartum reproductive variables such as first postpartum artificial insemination (AI) and pregnancy rate at 150 DIM were recorded.

### 2.9. Statistical Analysis

The statistical analysis was completed using SAS software package version 9.4 (SAS Institute Inc., Cary, NC, USA). Time series data for blood sampling outcome, BCS, and weekly milk yield and milk composition were analyzed using MIXED for repeated measure procedure with autoregressive covariance structure. Data were reported as least square means ± standard error of the mean, and the treatment comparison was completed by using the Tukey adjustment for multiple comparisons. *p*-values > 0.05 and ≤ 0.10 were considered a tendency, whereas *p*-values < 0.05 was defined to be statistically significant. Fisher’s exact test were used to investigate the effect of SeE and CZM on postpartum health, disorders, and reproductive performances. Cows without the first artificial insemination (%) over a period of 300 days from calving were analyzed by Kaplan–Meier survival analysis.

## 3. Results

### 3.1. Temperature–Humidity Index of Barn

During the experiment, the barn temperature was 15.5–35.5 °C, and the humidity was between 53–98%. The THI value of the experiment period was between 57.9–95.0, and the average THI value was 78.3.

### 3.2. Hematologic and Biochemical Profile Examination

The treatment means and *p* values of the main effects and interactions for hematological parameters were presented in Table 3. There was no significant treatment × time interactions for WBC (*p* = 0.78), RBC (*p* = 0.10), neutrophil (*p* = 0.99), lymphocytes (*p* = 0.99), and monocyte (*p* = 0.83). There was no time effect on WBC, neutrophils, lymphocytes, or monocytes, and there was a significant time effect for RBC (*p* < 0.001). There was no overall treatment effect (*p* > 0.05) on serum WBC and RBC levels. For neutrophils, there was a trend (*p* < 0.1) in CON higher than SeE and CZM cows prepartum. For monocytes, there was a trend (*p* < 0.1) in CON lower than SeE and CZM cows at d-14 and d-21. Monocytes in SeE and CZM cows were both significantly higher than in the CON cows at d7 (*p* < 0.05). HCT, HGB, MCV, MCH, MCHC, eosinophils, and basophils data were provided in Table S1.

The treatment means and *p* values of the main effects and interactions for blood biochemical profiles were presented in Table 4. There was no significant treatment × time interactions for albumin (*p* = 0.22), globulin (*p* = 0.84), and Ca (*p* = 0.61). There was no time effect on albumin, Ca and there was a significant time effect on globulin (*p* < 0.001). In all sampling days, there was no treatment effect (*p* > 0.05) on serum albumin, globulin, and A/G level. Higher (*p* < 0.05) Ca concentration was observed at d21 and d14 in the CZM group compared to CON. GOT, GPT, GGT, LDF, total protein, A/G, BUN, creatinine, triglyceride, CPK, P, Mg, Na, K, and Cl data were provided in Table S2. Since Ca status is mainly determined by dietary Ca content, phosphorus content, and cation–anion balance, the higher Ca concentration in the CZM group cannot be caused by trace mineral supplementation alone.

**Figure 1:**
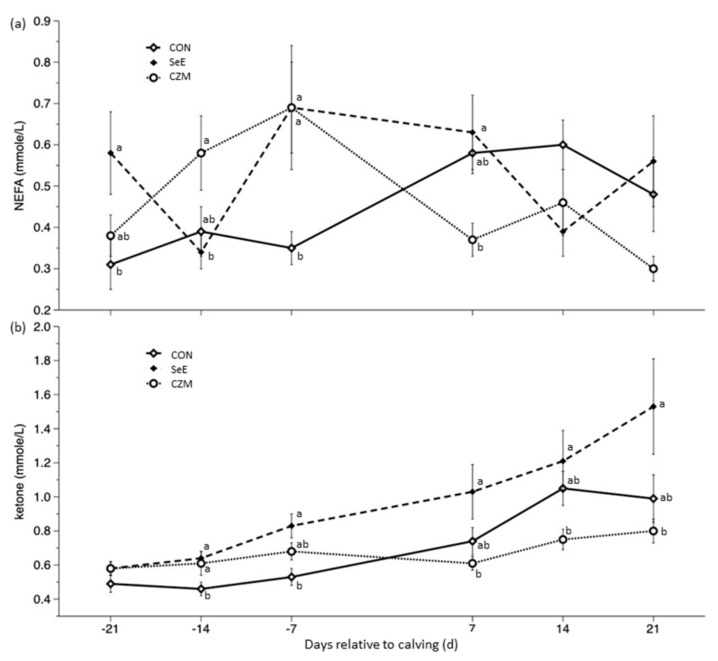
Effects of Se-vitamin E (SeE) or Cu-Zn-Mn (CZM) supplementation on (**a**) NEFA (time *p* = 0.13, treat × time *p* < 0.001) and (**b**) blood ketone (time *p* < 0.001, treat × time *p* < 0.01) changes from 21 days prepartum to 21 days postpartum in transition dairy cows. CON: fed a basal diet without supplementation, *n* = 8; SeE: supplemented with 1500 IU vitamin E and 0.3 ppm of organic Se, *n* = 8; CZM: supplemented with 12.5 ppm of organic Zn, 5 ppm of organic Cu, and 7.5 ppm Mn, *n* = 8. Values are means ± standard error of mean. ^a,b^, Values within the same time point with different superscripts differ significantly (*p* < 0.05).

Results of energy status indicators such as glucose, BHBA, and NEFA, were presented in Table 4, Figure 1a,b. There was no significant treatment × time interaction for glucose (*p* = 0.18), but significant for NEFA (*p* < 0.001) and BHBA (*p* < 0.01). There was no time effect on NEFA, but there was a significant time effect for glucose (*p* < 0.01) and ketone (*p* < 0.001). In all sampling days, there was no treatment effect (most *p* > 0.1) on glucose. The NEFA concentration of CON was lower than CZM or SeE group during d-21 to d7 (*p* < 0.05). In all sampling days except for d-21, higher (*p* < 0.05) BHBA concentration was observed in the SeE group. The peak concentrations of BHBA were recorded at d21.

### 3.3. Postpartum Health, Disorders, and BCS

The treatment effects on postpartum health, disorders category, and BCS change were presented in Table 5. All experiment cows had normal births without twins, miscarriages, or stillbirths. The RFM in the CON, SeE, and CZM was 25.0%, 0.0%, and 12.5%, respectively (*p* = 0.03). The subclinical hypocalcemia in the CON, SeE, and CZM was 87.5%, 41.7%, and 29.2%, respectively (*p* < 0.001). The subclinical ketosis in the CON, SeE, and CZM was 20.8%, 29.2%, and 8.3%, respectively (*p* = 0.19). The positive energy balance in the CON, SeE, and CZM was 20.8%, 29.2%, and 37.5%, respectively (*p* = 0.45). BCS change from calving to 21 d postpartum in the CON, SeE, and CZM was 0.16, 0.16, and 0.13, respectively (*p* = 0.86).

### 3.4. Milk Production and Composition

**Figure 2:**
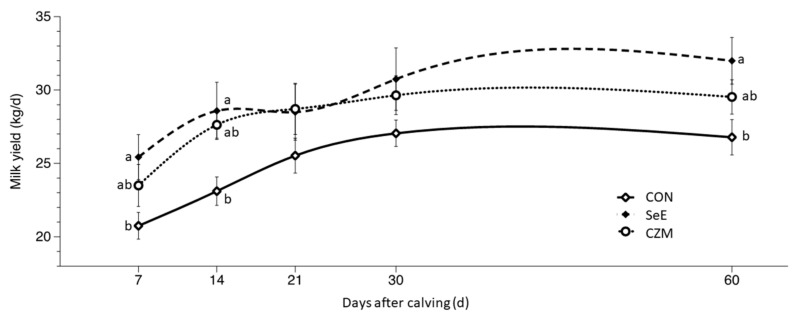
Effects of Se-vitamin E (SeE) or Cu-Zn-Mn (CZM) supplementation on milk yield from 7 to 60 days after calving in transition dairy cows. CON: fed a basal diet without supplementation, *n* = 8; SeE: supplemented with 1500 IU vitamin E and 0.3 ppm of organic Se, *n* = 8; CZM: supplemented with 12.5 ppm of organic Zn, 5 ppm of organic Cu and 7.5 ppm Mn, *n* = 8. Values are means ± standard error of mean. ^a,b^, Values within the same time point with different superscripts differ significantly (*p* < 0.05). Time *p* < 0.001, treat × time *p* = 0.37.

Treatment effects for milk production and composition were presented in Table 6 and Figure 2. Treatment effects (*p* < 0.05) were observed for milk yield due to greater responses in SeE than CZM or CON at d7, d14, and d60. SCLS was significantly (*p* < 0.05) lower in CZM than SeE or CON at d14 and d30, and a trend (*p* = 0.1) for lower SCLS in CZM was detected at d60. MFP was significantly (*p* < 0.05) higher in SeE than CZM or CON at d60. There were no treatment effects (*p* > 0.05) on MPP. There was greater (*p* < 0.05) MLP in CZM. MUN was significantly (*p* < 0.05) lower in CON than SeE or CZM at d14, d30, and d60. MLP, MUN, MSNFP, MTSP, SCC, and MC were provided in Table S3.

### 3.5. Reproductive Performance

**Figure 3:**
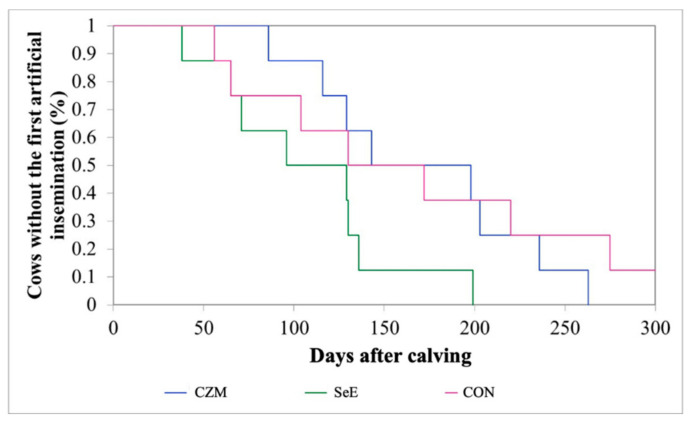
Kaplan–Meier survival analysis of the first artificial insemination (%) over a period of 300 days from calving in this study. CZM: supplemented with 12.5 ppm of organic Zn, 5 ppm of organic Cu, and 7.5 ppm Mn, *n* = 8; SeE: supplemented with 1500 IU vitamin E and 0.3 ppm of organic Se, *n* = 8; CON: fed a basal diet without supplementation, *n* = 8. The *p*-values between the groups were 0.49 for CON and CZM, 0.01 for CON and SeE, and 0.003 for SeE and CZM.

All of the experiment cows were without abortion and had vagina prolapse during gestation and dystocia at the calving time. The mean interval from calving to the first AI postpartum in the CON, SeE, and CZM was 162, 108, and 171 days, respectively. The K-M survival curve showed that 50% of cows in the SeE resumed the first AI by 100 days (Figure 3). The *p* values between the groups were 0.49 for CON and CZM, 0.01 for CON and SeE, and 0.003 for SeE and CZM. The results showed that SeE had the shortest interval from postpartum to the first AI. The pregnancy rates on the 150th day postpartum of the CON, SeE, and CZM were 25.0%, 37.5%, and 25.0%, respectively (*p* = 0.82) (Table 5).

## 4. Discussion

When the THI is greater than 72, this indicates the dairy cow is exposed to a state of heat stress [23], when the THI value is between 80 to 89, it means the cow is in a state of moderate heat stress [24]. The THI value in this study was between 57.9–95.0, the dairy cows were in a state of moderate heat stress for a long period of time (about 42 days). During the experimental period, the barn had been equipped with a cooling system including fans and water spray. Heat stress has been one of the most concerning stressors by the dairy industry, as milk production, reproductive performance, and health are all sensitive to heat stress [25]. Prepartum heat exposure also suppresses immune function [26]. Dairy cows exposed to heat stress during lactation, a 40% loss of milk yield and a 20–30% reduction in conception rate are common [27]. In this study, milk yield losses of 16% and 9% in CON compared to SeE and CZM were observed at d60, respectively. Conception rate declines of 12.5% in CON compared to SeE, and there were no different between CON and CZM in 150 DIM. Nutrient intake can only explain part of heat stress effects in cattle [28]. Heat stress results in suppressed gonadotrophin secretion and inhibition of reproduction [29]. In this study, higher milk production of SeE and CZM groups indicated that the supplement of trace elements and vitamin E may enable cows to better adaption to heat stress. Supplementation with Se, Cu, Co, I, Zn, vitamin E, and vitamin A had been reported to have beneficial effects on heat-stressed transition dairy cows [30].

Neutrophils constitute the first line of after calving defense against the invading pathogens in the uterine lumen [31]. Before and at parturition, the number of blood lymphocytes declined and returned to a higher level during the second week postpartum [32]. In this study, there is a trend in the CON group showing neutrophil populations increasing and lymphocytes decreasing within 2 wks before calving (*p* < 0.1, Table 3). Stress and neuroendocrine alters both have a direct effect on the neutrophile and lymphocyte counts during the peripartum period [33]. Although there was no significant difference in neutrophile and lymphocyte counts among the three groups in the present study, cows of CZM did show significantly lower SCLS than CON which indicated there was a difference in immune function in the mammary gland. In addition, the immunosuppression of transition cows due to impaired proliferation of lymphocytes, phagocytic activity of the neutrophils, leukocyte function, and bactericidal efficiency will increase the uterine and endometritis infection risk [34]. Cows of SeE had a lower incidence of RFM (Table 5) which will lower the risk of endometritis compared to CON. Cattle diagnosed with metritis in the first 65 d postpartum had a lower first-service pregnancy rate [35]. Metritis may be caused by factors, such as fetal maceration, retained placentas, or dystocia [36]. RFM could raise the incidence of diseases such as metritis, cystic ovaries, and low fertility [37]. Supplementation with SeE can reduce mastitis, RFM, days to estrus, and abortion in dairy cows [38]. Lowering the incidence of RFM in SeE might have a positive influence on reproductive performance. In this study, supplementation of Se and vitamin E during the transition period shorten the days between the first AI and calving (*p* < 0.05). The SeE had significantly better reproductive performance (*p* < 0.05). A strong negative correlation between blood concentrations of Zn and RFM incidence was noted in early post-calving cows [39]. In our study, CZM was not as effective as SeE in reducing the incidence of RFM. The immune system creates an appropriate microenvironment to establish the first stage of pregnancy [4]. In this study, SeE supplementation improved immune function and achieved better reproductive performance. Hypocalcemia could increase the risk for RFM [40]. Similarly, the blood Ca concentrate of CON was below the reference value during the experiment period and it was the lowest among the three groups at d14 and d21. The incidence of RFM in CON was higher than in SeE.

High serum concentrations of NEFA, BHBA, and low concentrations of Ca around parturition were associated with early lactation milk loss, and low Ca concentration around parturition may render impaired reproduction in the early lactation period [41]. If cows are incapable to change their energy metabolism rapidly enough to support milk synthesis, they either produce milk below their capability or are susceptible to metabolic disorders [41]. Ca concentration of CON was below the reference range, and it was significantly lower than CZM at d14 and d21. In addition, there was the lowest milk yield in CON at d14 and d60 (*p* < 0.05). Cows with elevated NEFA within 2 wk before calving and with elevated NEFA and BHBA within 2 wk after calving are less likely to become pregnant in early lactation [42]. According to the NEFA data of the present study, the interacting effect was significant (*p* < 0.05). The result indicated that the effect of supplementing SeE and CZM on NEFA changed with time during transition period. The interacting effect was also significant (*p* < 0.05) on BHBA. CZM treatment lowers postpartum NEFA and BHBA. The concentration of BHBA in the SeE group continues to rise. The SeE group with a circulating concentration of BHBA ≥ 1.2 mmol/L at d14 and d21 were considered having subclinical ketosis. Although, the concentration of NEFA and BHBA in SeE was significantly higher than other groups and BHBA reached peak at d21, milk production and reproductive performance of SeE was the best. It is possible that SeE increased milk yield by lowering oxidative stress and improved reproductive performance by prevention of RFM which in turn lowered the risk of endometritis. However, the SeE could not prevent the negative energy balance situation. The high milk production in the SeE group might worsen the negative energy balance situation.

The greatest proportion of postpartum disorders fell into the CON group since no preventive measure was offered for both inflammatory and metabolic transition diseases. Both inflammatory and metabolic transition diseases are associated with increased NEFA [43]. SeE supplementation in the pre-calving period can increase immune responses, and antioxidant capacity after parturition and reduce mastitis and RFM in dairy cows, low SCC, and enhance mammary health [38,44]. RFM decreased feed intake which in turn lowered milk yield [45]. In this study, the significantly lower incidence of RFM, and greater milk yield in SeE cows at d60, BHBA, and NEFA were higher than CON at some time point. These results showed that supplementing SeE could reduce inflammation in transition dairy cattle and prevent RFM which indirectly had a positive influence on milk production and reproduction performance, but it did not help improve energy balance. Substantial evidence exists that trace minerals such as Zn, Cu, and Mn can minimize the negative effects of oxidative state [46]. Higher rates of metritis, mastitis, and RFM were declared to be associated with lower Zn nutritional status [47]. SCLS in CZM was significantly lower than CON at d14 (Table 6). In addition, SCLS in CZM tended to be lower at d30 and d60 (*p* < 0.1).

## 5. Conclusions

In this study, the supplementation of trace elements and vitamin E may enable cows in SeE and CZM to better adapt to heat stress. SeE supplementation improved immune function, reproductive performance, and milk yield, but not negative energy balance status as shown in higher blood NEFA and BHBA concentrations. Supplementation of CZM improved milk yield and energy regulation through antioxidative capacity and immune function but had no influence on reproductive performance.

## Figures and Tables

**Table 1:** Ingredients of concentrate and total mixed ration used in the feeding of experiment cows in prepartum or postpartum periods.

Item	Concentrate Prepartum (% Dry Matte Basis)	Total Mixed Ration (% Dry Matte Basis)
Corn silage	-	17.7
Bermuda grass hay	-	18.0
Alfalfa hay	-	4.5
Brewers grains, wet	-	10.5
Soybean hull	-	21.0
Wheat bran	-	13.3
Corn	60.0	7.37
Corn gluten meal	-	1.05
Soybean meal, 44% CP	31.3	4.42
Fish meal	-	0.52
Molasses	5.0	0.75
Iodized salt	1.0	0.12
Sodium bicarbonate	-	0.31
Limestone	1.0	0.27
Calcium diphosphate	0.8	-
Premix ^†^	0.5	0.18
Mineral–vitamin mix ^‡^	-	0.01
Total	100	100

^†^ Each kilogram of premix has Vit. A, 10,000,000 IU; Vit. D_3_, 1,600,000 IU; Vit. E, 70,000 IU; Fe, 50 g; Cu, 10 g; Zn, 40 g; I, 0.5 g; Se, 0.1 g; Co, 0.1 g. ^‡^ Each kilogram of premix has Vit. A, 1,200,000 IU; Vit. D, 700,000 IU; Vit. E, 1500 IU; Fe, 5 g; Cu, 1 g; Zn, 8 g; I, 0.5 g; Se, 0.1 g; Co, 0.1 g; Mn, 3.5 g; Ca, 0.07 g; Na, 0.12 g; Mg, 0.07 g.

**Table 2:** Nutrient composition of concentrate, pangola grass hay, and total mixed ration used in the feeding of experiment cows in prepartum or postpartum periods.

Nutrient Composition	Concentrate Prepartum	Total Mixed Ration	Pangola Grass Hay
Dry matter, %	89.9	45.9	92.9
Crude protein, %	17.1	14.6	4.6
Fat, %	2.3	2.3	1.7
Neutral detergent fiber, %	23.4	45.4	72.8
Acid detergent fiber, %	11.9	28.1	43.9
Ca, %	0.7	0.6	0.17
P, %	0.5	0.4	0.38
NE_l_ Mcal/kg	1.5	1.6	1.25
Cu, ppm of DM	16.0	8.0	5.0
Mn, ppm of DM	-	0.2	164.0
Se, ppm of DM	0.16	0.2	-
Zn, ppm of DM	64.0	33.0	17.0

**Table 3:** Effects of Se-vitamin E (SeE) or Cu-Zn-Mn (CZM) supplementation on hematologic profile changes from 21 days prepartum to 21 days postpartum in transition dairy cows.

Item	Treatment	Days after Calving					
		−21	−14	−7	7	14	21
WBC	SeE	13,276.3	13,012.5	12,277.5	12,262.5	13,468.8	13,568.8
(1/uL)	CZM	11,850.0	11,988.8	11,553.8	9776.3	10,725.0	11,065.0
	CON	11,002.5	11,401.3	11,252.5	9737.5	9875.0	10,887.5
	SEM	2654.18	2677.54	1681.69	2706.02	3478.86	3050.03
	treatment *p* value	0.23	0.50	0.48	0.10	0.09	0.15
	time *p* value	0.07					
	treat × time *p* value	0.78					
RBC	SeE	5.6	5.8	5.8	5.8	5.4	5.3
(10^6^/uL)	CZM	6.1	6.1	6.1	5.9	5.5	5.4
	CON	5.8	5.7	5.8	5.6	5.2	4.4
	SEM	0.62	0.74	0.61	0.62	0.57	1.10
	treatment *p* value	0.29	0.51	0.51	0.56	0.63	0.12
	time *p* value	<0.001					
	treat × time *p* value	0.10					
Neutrophils	SeE	28.3	29.5	34.2	31.4	33.6	31.4
(%)	CZM	32.8	34.6	35.6	36.2	35.2	37.0
	CON	38.0	38.0	41.9	40.2	38.4	36.4
	SEM	9.07	7.28	7.05	12.72	11.91	13.61
	treatment *p* value	0.10	0.06	0.06	0.41	0.73	0.68
	time *p* value	0.54					
	treat × time *p* value	0.99					
Lymphocytes	SeE	62.08	61.66	57.31	58.50	56.54	59.89
(%)	CZM	57.49	56.49	55.18	53.48	55.05	53.53
	CON	53.08	52.77	49.01	50.06	51.88	53.93
	SEM	9.11	8.18	7.61	12.51	11.95	13.69
	treatment *p* value	0.14	0.09	0.07	0.42	0.75	0.60
	time *p* value	0.45					
	treat × time *p* value	0.99					
Monocytes	SeE	6.69	5.75	5.64	6.98 ^a^	6.76	6.43
(%)	CZM	6.84	6.41	6.25	7.07 ^a^	6.53	6.55
	CON	5.86	5.27	6.01	6.24 ^b^	6.79	7.03
	SEM	1.63	1.78	1.31	0.66	0.98	1.82
	treatment *p* value	0.45	0.46	0.66	0.01	0.85	0.80
	time *p* value	0.11					
	treat × time *p* value	0.83					

Abbreviations: WBC, white blood cell count; RBC, red blood cell count; SeE: supplemented with 1500 IU vitamin E and 0.3 ppm of organic Se, *n* = 8; CZM: supplemented with 12.5 ppm of organic Zn, 5 ppm of organic Cu and 7.5 ppm Mn, *n* = 8; CON: fed a basal diet without supplementation, *n* = 8. SEM, standard error of mean. ^a,b^, Values of each measurement within the same column with different superscripts differ significantly (*p* < 0.05). Reference value: WBC 4000–12,000 (1/uL); RBC 5–10 (10^6^/uL); neutrophils 15–33 (%); lymphocytes 60–62.5 (%); monocytes 0.75–7 (%) [22].

**Table 4:** Effects of Se-vitamin E (SeE) or Cu-Zn-Mn (CZM) supplementation on biochemical profile and glucose changes from 21 days prepartum to 21 days postpartum in transition dairy cows.

Item	Treatment	Days after Calving					
		−21	−14	−7	7	14	21
Albumin	SeE	3.95	3.65	3.74	3.41	3.61	3.85
(g/dL)	CZM	3.75	3.68	3.49	3.64	3.68	3.69
	CON	3.54	3.59	3.59	3.77	3.42	3.57
	SEM	0.45	0.47	0.35	0.48	0.55	0.35
	treatment *p* value	0.20	0.93	0.38	0.33	0.65	0.28
	time *p* value	0.52					
	treat × time *p* value	0.22					
Globulin	SeE	3.08	2.96	2.92	3.09	3.54	3.64
(g/dL)	CZM	3.06	2.74	2.99	3.13	3.56	3.80
	CON	3.34	3.10	2.89	3.38	3.45	3.74
	SEM	0.55	0.47	0.42	0.54	0.58	0.60
	treatment *p* value	0.54	0.33	0.89	0.53	0.93	0.87
	time *p* value	<0.001					
	treat × time *p* value	0.84					
Ca	SeE	7.99	8.14	7.92	8.00	8.38 ^ab^	8.54 ^ab^
(mg/dL)	CZM	8.16	7.93	8.22	8.06	8.58 ^a^	8.64 ^a^
	CON	7.35	7.60	7.51	7.61	7.40 ^b^	7.60 ^b^
	SEM	0.93	0.79	0.70	0.84	0.93	0.88
	treatment *p* value	0.19	0.40	0.12	0.53	0.02	0.03
	time *p* value	0.07					
	treat × time *p* value	0.61					
Glucose	SeE	57.8	53.0	58.0	50.9	49.5	56.6
(mg/dL)	CZM	56.4	52.9	57.9	52.7	52.6	59.8
	CON	58.9	58.4	56.6	54.5	56.8	55.3
	SEM	5.12	11.98	3.90	8.57	9.44	6.22
	treatment *p* value	0.38	0.35	0.54	0.50	0.09	0.11
	time *p* value	<0.01					
	treat × time *p* value	0.18					

Abbreviations: SeE: supplemented with 1500 IU vitamin E and 0.3 ppm of organic Se, *n* = 8; CZM: supplemented with 12.5 ppm of organic Zn, 5 ppm of organic Cu and 7.5 ppm Mn, *n* = 8; CON: fed a basal diet without supplementation, *n* = 8. SEM, standard error of mean. ^a,b^, Values of each measurement within the same column with different superscripts differ significantly (*p* < 0.05). Reference value: albumin 2.1–3.6 (g/dL); globulin 3–4.9 (g/dL); Ca 9.7–12.4 (mg/dL); glucose 45–75 (mg/dL) [22].

**Table 5:** Postpartum BCS change, health, or disorders category and pregnancy rates of experiment cows at 150th day postpartum.

Item ^1^	Treatment ^2^			
	CON	SeE	CZM	*p*-Value
BCS loss from calving to 21 d	0.16	0.16	0.13	0.86
Retained fetal membrane (%)	25.0 ^b^	0.0 ^a^	12.5 ^ab^	0.03
Subclinical hypocalcemia (%)	87.5 ^b^	41.7 ^a^	29.2 ^a^	<0.001
Subclinical ketosis (%)	20.8	29.2	8.3	0.19
Positive energy balance (%)	20.8	29.2	37.5	0.45
Pregnancy rates at 150th day postpartum (%)	25.0	37.5	25.0	0.82

^1^ Abbreviations: BCS, body condition score, BCS change from calving to 21 days postpartum in transition dairy cows. ^2^ CON: fed a basal diet without supplementation, *n* = 8; SeE: supplemented with 1500 IU vitamin E and 0.3 ppm of organic Se, *n* = 8; CZM: supplemented with 12.5 ppm of organic Zn, 5 ppm of organic Cu, and 7.5 ppm Mn, *n* = 8. ^a,b^, Values of each measurement within the same row with different superscripts differ significantly (*p* < 0.05).

**Table 6:** Effects of Se-vitamin E (SeE) or Cu-Zn-Mn (CZM) supplementation on milk yield and composition.

Item	Treatment	Days after Calving					
		7	14	21	30	60	305
Milk yield (kg)	SeE	25.4 ^a^	28.6 ^a^	28.5	30.7	32.0 ^a^	8811.6
	CZM	23.5 ^ab^	27.6 ^ab^	28.7	29.6	29.5 ^ab^	8974.8
	CON	20.8 ^b^	23.1 ^b^	25.5	27.1	26.8 ^b^	8684.5
	SEM	5.51	5.85	6.59	6.23	5.65	1203.3
	Treatment *p* value	0.05	0.02	0.32	0.23	0.03	0.90
	Time *p* value	<0.001					
	Treat × time *p* value	0.37					
SCLS	SeE	5.08	3.66 ^ab^	3.35	2.96	3.34	
	CZM	4.65	2.65 ^b^	3.12	2.17	2.39	
	CON	5.86	4.90 ^a^	4.24	3.59	4.43	
	SEM	1.81	1.72	2.04	1.68	1.83	
	Treatment *p* value	0.41	0.05	0.54	0.26	0.1	
	Time *p* value	<0.001					
	Treat × time *p* value	0.99					
MFP (%)	SeE	4.60	3.93	3.92	3.62	4.34 ^a^	
	CZM	4.13	3.71	3.64	3.75	3.86 ^ab^	
	CON	4.80	4.20	4.01	3.63	3.50 ^b^	
	SEM	0.99	0.67	0.65	0.64	0.85	
	Treatment *p* value	0.14	0.11	0.25	0.82	0.02	
	Time *p* value	<0.001					
	Treat × time *p* value	<0.01					
MPP (%)	SeE	3.80	3.29	2.84	2.81	2.87	
	CZM	3.83	3.18	2.95	2.90	2.99	
	CON	3.62	3.14	2.89	2.83	2.90	
	SEM	0.45	0.25	0.24	0.23	0.21	
	Treatment *p* value	0.38	0.20	0.42	0.56	0.25	
	Time *p* value	<0.001					
	Treat × time *p* value	0.18					

Abbreviations: SCLS, somatic cell linear score; MFP, milk fat percentage; MPP, milk protein percentage; 305, 305-2X-ME. SeE: supplemented with 1500 IU vitamin E and 0.3 ppm of organic Se, *n* = 8; CZM: supplemented with 12.5 ppm of organic Zn, 5 ppm of organic Cu, and 7.5 ppm Mn, *n* = 8; CON: fed a basal diet without supplementation, *n* = 8. SEM, standard error of mean. ^a,b^, Values of each measurement within the same column with different superscripts differ significantly (*p* < 0.05).

## Data Availability

Data are contained within the article.

## Supplementary Materials

The following supporting information can be downloaded at: https://www.mdpi.com/article/10.3390/vetsci10030225/s1, Table S1: Effects of SeE or CZM supplementation on HCT, hemoglobin, mean corp. vol., mean corp. hgb conc., eosinophils and basophils data changes in transition dairy cattle. Table S2: Effects of SeE or CZM supplementation on total protein, urea nitrogen, creatinine, triglyceride, creatine kinase, P, Mg, Na, K, and Cl data changes in transition dairy cattle. Table S3: Effects of SeE or CZM supplementation on somatic cell count data changes in transition dairy cattle.
