# Supplementary material for: Effect of Supplementing Vitamin E, Selenium, Copper, Zinc, and Manganese during the Transition Period on Dairy Cow Reproductive Performance and Immune Function
Source: Vet Sci. 2023 Mar 15;10(3):225. doi: 10.3390/vetsci10030225 (PMC10059820; doi:10.3390/vetsci10030225)
Supplement: Supplementary file 1 [file vetsci-10-00225-s001.zip › vetsci-2202144-supplementary.pdf]

Table S1. Effects of SeE or CZM supplementation on WBC, RBC, HCT, HGB, MCV, MCH, eosinophils, basophils and monocyte data changes in transition dairy cattle.

**Item**[1]	Treatment[2]	Days after calving					
		-21	-14	-7	7	14	21
**WBC**	SeE	13276.3	13012.5	12277.5	12262.5	13468.8	13568.8
(1/ul)	CZM	11850.0	11988.8	11553.8	9776.3	10725.0	11065.0
	CON	11002.5	11401.3	11252.5	9737.5	9875.0	10887.5
	SEM	2654.18	2677.54	1681.69	2706.02	3478.86	3050.03
	treatment *p* value	0.23	0.50	0.48	0.10	0.09	0.15
	time *p* value	0.07					
	treat × time *p* value	0.78					
RBC	SeE	5.6	5.8	5.8	5.8	5.4	5.3
(10^6/ul)	CZM	6.1	6.1	6.1	5.9	5.5	5.4
	CON	5.8	5.7	5.8	5.6	5.2	4.4
	SEM	0.62	0.74	0.61	0.62	0.57	1.10
	treatment *p* value	0.29	0.51	0.51	0.56	0.63	0.12
	time *p* value	< 0.001					
	treat × time *p* value	0.10					
HCT	SeE	30.1	31.2	31.8	31.0	28.3	27.2
(%)	CZM	31.8	32.0	32.1	30.4	27.7	27.2
	CON	31.1	30.7	30.9	29.4	26.8	25.1
	SEM	2.80	3.41	3.21	2.86	2.50	2.76
	treatment *p* value	0.50	0.78	0.75	0.53	0.51	0.20
	time *p* value	< 0.001					
	treat × time *p* value	0.55					
HGB	SeE	9.6	9.7	9.9	9.7	8.9	8.9
(g/dl)	CZM	9.8	9.8	9.9	9.4	8.6	8.5
	CON	9.6	8.4	9.5	9.0	8.3	8.1
	SEM	0.77	2.14	1.00	0.93	0.82	1.00
	treatment *p* value	0.83	0.36	0.72	0.30	0.36	0.29
	time *p* value	< 0.001					
	treat × time *p* value	0.72					
MCV	SeE	54.1	54.6	55.5	53.9	53.0	51.9
(fL)	CZM	52.3	52.4	53.0	51.4	51.0	50.7
	CON	53.2	53.4	53.5	52.6	51.7	51.6
	SEM	4.36	4.40	4.06	4.33	4.12	3.65
	treatment *p* value	0.72	0.65	0.46	0.55	0.64	0.81
	time *p* value	< 0.001					
	treat × time *p* value	0.86					
MCH	SeE	16.0	17.0	17.3	16.9	16.7	16.9
(pg)	CZM	16.1	16.1	16.4	16.0	15.8	15.8
	CON	16.5	16.6	16.6	16.1	16.0	16.6
	SEM	2.07	1.56	1.39	1.59	1.44	1.81
	treatment *p* value	0.91	0.55	0.37	0.44	0.44	0.49
	time *p* value	0.28					
	treat × time *p* value	0.74					
MCHC	SeE	31.9	31.1	31.2	31.3	31.5	32.5
(%)	CZM	30.8	30.7	30.9	31.0	30.9	31.2
	CON	31.0	31.1	30.9	30.6	31.0	32.2
	SEM	1.47	0.78	0.51	0.82	0.58	2.21
	treatment *p* value	0.35	0.59	0.43	0.19	0.11	0.49

	time *p* value	0.03					
	treat × time *p* value	0.89					
Eosinophils (%)	SeE	2.2	2.4	2.2	2.5	2.4	1.9
	CZM	2.1	1.9	2.1	2.6	2.4	2.2
	CON	2.3	2.7	2.5	2.9	2.3	2.2
	SEM	0.64	0.93	0.75	0.62	0.66	0.71
	treatment *p* value	0.83	0.19	0.56	0.42	0.88	0.74
	time *p* value	0.06					
	treat × time *p* value	0.70					
Basophils (%)	SeE	0.7	0.7	0.7	0.6	0.7	0.6
	CZM	0.8	0.6	0.8	0.7	0.9	0.8
	CON	0.7	0.6	0.6	0.8	0.7	0.5
	SEM	0.25	0.19	0.28	0.27	0.21	0.29
	treatment *p* value	0.66	0.52	0.17	0.59	0.09	0.11
	time *p* value	0.48					
	treat × time *p* value	0.27					
Monocyte (%)	SeE	6.69	5.75	5.64	6.98[a]	6.76	6.43
	CZM	6.84	6.41	6.25	7.07[a]	6.53	6.55
	CON	5.86	5.27	6.01	6.24[b]	6.79	7.03
	SEM	1.63	1.78	1.31	0.66	0.98	1.82
	treatment *p* value	0.45	0.46	0.66	0.01	0.85	0.80
	time *p* value	0.11					
	treat × time *p* value	0.83					

[1] WBC, white blood cell count; RBC, red blood cell count; HCT, hematocrit; HGB, hemoglobin; MCV, mean corpuscular volume; MCH, mean corpuscular hemoglobin; MCHC, mean corpuscular hemoglobin concentrate.

[2]SeE: supplemented with 1,500 IU vitamin E and 0.3 ppm of organic Se, n = 8; CZM: supplemented with 12.5 ppm of organic Zn, 5 ppm of organic Cu and 7.5 ppm Mn, n = 8; CON: fed a basal diet without supplementation, n = 8. SEM, standard error of mean.

[ab], Values within a column with different superscripts differ treatment effect significantly ($P < 0.05$).

Reference value: WBC 4000-12000 (1/ul); RBC 5-10 (10^6/ul); HCT 24-146 (%); HGB 8-15 (g/dL); MCV 40-60 (fL); MCH 11-17 (pg); MCHC 30-36 (%); eosinophils 0-20 (%); basophils 0-1.6 (%); monocyte 0.75-7 (%) [22].

Table S2. Effects of SeE or CZM supplementation on GOT, GPT, GGT, LDH, total protein, albumin, globulin, A/G, BUN, creatinine, triglyceride, CPK, P, Mg, Na, K and Cl data changes in transition dairy cattle.

**Item**[1]	Treatment[2]	Days after calving					
		-21	-14	-7	7	14	21
**GOT**	SeE	62.9	60.4	64.6	84.6	89.2	91.6
(U/L)	CZM	74.0	68.9	71.8	95.1	94.7	86.9
	CON	65.7	63.1	71.3	93.8	92.8	91.2
	SEM	11.27	8.71	9.91	17.04	20.93	20.01
	treatment *p* value	0.12	0.14	0.29	0.43	0.88	0.88
	time *p* value	< 0.001					
	treat × time *p* value	0.82					
GPT	SeE	24.9	23.0	20.4	19.4	22.0	26.1
(U/L)	CZM	23.3	20.1	21.3	18.1	22.3	21.9
	CON	25.0	25.1	22.8	22.6	23.1	24.4
	SEM	5.61	5.96	3.62	5.71	4.65	5.14
	treatment *p* value	0.80	0.24	0.46	0.27	0.90	0.27
	time *p* value	< 0.01					
	treat × time *p* value	0.66					
GGT	SeE	15.6	13.4	14.4	15.4	14.6	21.0
(U/L)	CZM	14.8	15.0	14.0	16.1	15.2	19.3
	CON	14.1	14.3	15.2	14.9	17.7	18.3
	SEM	3.07	3.58	3.12	3.58	6.55	6.17
	treatment *p* value	0.63	0.71	0.77	0.80	0.62	0.69
	time *p* value	< 0.001					
	treat × time *p* value	0.77					
LDH	SeE	1849.3[b]	1743.3[b]	1722.6	1891.8	1942.8	2249.1[a]
(U/L)	CZM	2154.3[a]	2055.8[a]	1988.0	1971.0	2020.5	2092.2[ab]
	CON	1799.3[b]	1742.3[b]	1852.7	1776.7	1924.6	1827.0[b]
	SEM	265.04	247.07	251.92	269.86	255.72	300.99
	treatment *p* value	0.01	0.01	0.11	0.37	0.75	0.01
	time *p* value	< 0.01					
	treat × time *p* value	0.01					
Total Protein	SeE	7.03	6.61	6.66	6.62	7.14	7.49
(g/dL)	CZM	6.94	6.42	6.49	6.66	7.24	7.51
	CON	6.88	6.69	6.47	7.15	6.87	7.31
	SEM	0.68	0.73	0.58	0.53	0.68	0.55
	treatment *p* value	0.92	0.78	0.79	0.07	0.54	0.75
	time *p* value	< 0.001					
	treat × time *p* value	0.32					
Albumin	SeE	3.95	3.65	3.74	3.41	3.61	3.85
(g/dL)	CZM	3.75	3.68	3.49	3.64	3.68	3.69
	CON	3.54	3.59	3.59	3.77	3.42	3.57
	SEM	0.45	0.47	0.35	0.48	0.55	0.35
	treatment *p* value	0.20	0.93	0.38	0.33	0.65	0.28
	time *p* value	0.52					
	treat × time *p* value	0.22					
Globulin	SeE	3.08	2.96	2.92	3.09	3.54	3.64
(g/dL)	CZM	3.06	2.74	2.99	3.13	3.56	3.80
	CON	3.34	3.10	2.89	3.38	3.45	3.74
	SEM	0.55	0.47	0.42	0.54	0.58	0.60
	treatment *p* value	0.54	0.33	0.89	0.53	0.93	0.87

	time p value	< 0.001					
	treat × time p value	0.84					
A/G	SeE	1.3	1.2	1.3	1.2	1.1	1.1
	CZM	1.2	1.3	1.2	1.1	1.0	1.0
	CON	1.1	1.1	1.1	1.1	1.0	1.0
	SEM	0.23	0.23	0.21	0.26	0.26	0.26
	treatment p value	0.19	0.15	0.37	0.97	0.97	0.42
	time p value	< 0.01					
	treat × time p value	0.72					
BUN (mg/dL)	SeE	6.0	7.6	7.9	13.9	15.1[a]	12.5
	CZM	6.9	6.2	8.0	11.2	12.3[ab]	14.2
	CON	6.9	5.4	7.4	11.1	10.7[b]	12.0
	SEM	2.07	2.70	2.13	3.44	3.34	3.03
	treatment p value	0.63	0.26	0.86	0.17	0.02	0.35
	time p value	< 0.001					
	treat × time p value	0.13					
Creatinine (mg/dL)	SeE	1.47	1.49	1.47	1.29	1.08	1.09
	CZM	1.52	1.50	1.51	1.12	1.06	1.01
	CON	1.42	1.42	1.46	1.25	1.25	1.18
	SEM	0.11	0.13	0.14	0.19	0.21	0.19
	treatment p value	0.15	0.44	0.71	0.21	0.14	0.23
	time p value	< 0.001					
	treat × time p value	0.02					
Triglyceride (mg/dL)	SeE	28.3	23.1	21.4	12.9	15.4	13.1
	CZM	25.4	19.7	18.4	13.7	12.2	10.9
-	CON	22.9	25.6	22.2	16.4	10.2	17.8
	SEM	7.12	7.12	5.41	7.18	8.26	7.49
	treatment p value	0.34	0.25	0.34	0.61	0.46	0.17
	time p value	< 0.001					
	treat × time p value	0.12					
CPK (U/L)	SeE	240.4	174.2	197.2	197.0	315.1	270.2
	CZM	219.0	227.5	241.3	267.8	259.1	248.9
	CON	166.2	170.8	183.2	211.9	173.8	216.7
	SEM	88.30	90.19	79.59	93.45	130.59	103.83
	treatment p value	0.23	0.39	0.33	0.29	0.09	0.60
	time p value	0.16					
	treat × time p value	0.41					
P (mg/dL)	SeE	6.20	6.54	6.64	5.54	6.46	6.06
	CZM	6.64	6.68	6.68	4.88	5.70	6.00
	CON	6.30	5.88	6.34	4.85	5.29	5.35
	SEM	1.10	1.43	1.01	1.18	1.29	1.14
	treatment p value	0.72	0.51	0.78	0.44	0.19	0.40
	time p value	< 0.001					
	treat × time P value	0.49					
Mg (mg/dL)	SeE	2.29	3.10	3.28	4.60	4.43	4.84
	CZM	2.39	2.43	2.82	2.37	5.41	4.75
	CON	3.33	2.35	2.55	3.73	3.80	3.83
	SEM	1.40	1.38	1.20	2.60	2.94	2.90
	treatment p value	0.27	0.51	0.50	0.23	0.56	0.76
	Time p value	< 0.001					
	treat × time p value	0.20					
Na	SeE	146.6	147.6	147.4	144.9	143.3	142.9

(mmol/L)	CZM	146.3	147.7	148.1	145.5	143.6	143.8
	CON	147.2	146.9	147.4	146.3	145.9	145.8
	SEM	1.37	3.16	2.37	2.38	3.38	2.58
	treatment *p* value	0.36	0.85	0.79	0.53	0.25	0.07
	time *p* value	< 0.001					
	treat × time *p* value	0.49					
K (mmol/L)	SeE	4.65	4.44	4.40	4.53	4.44	4.56
	CZM	4.71	4.53	4.56	4.46	4.69	4.38
	CON	4.58	4.41	4.49	4.53	4.50	4.40
	SEM	0.39	0.27	0.21	0.21	0.32	0.30
	treatment *p* value	0.80	0.70	0.36	0.81	0.28	0.41
	time *p* value	0.21					
	treat × time *p* value	0.79					
Cl (mmol/L)	SeE	107.0	106.6	107.7	104.3	103.0	102.6
	CZM	106.6	107.5	108.9	106.5	102.9	102.5
	CON	107.5	106.9	107.9	104.9	105.0	103.5
	SEM	2.263	3.393	2.641	2.498	3.609	2.643
	treatment *p* value	0.78	0.89	0.65	0.20	0.44	0.73
	time *p* value	< 0.001					
	treat × time *p* value	0.85					

[1]GOT, aspartate transaminase; GPT, alanine aminotransferase; GGT, γ-glutamyltransferase; LDH, lactate dehydrogenase; BUN, blood urea nitrogen; CPK, creatine phosphor kinase.

[2]SeE: supplemented with 1,500 IU vitamin E and 0.3 ppm of organic Se, n = 8; CZM: supplemented with 12.5 ppm of organic Zn, 5 ppm of organic Cu and 7.5 ppm Mn, n = 8; CON: fed a basal diet without supplementation, n = 8. SEM, standard error of mean.

[ab], Values within a column with different superscripts differ treatment effect significantly ($P < 0.05$).

Reference value: GOT 78-132 (U/L); GPT 11-40 (U/L); GGT 6.1-17.4 (U/L); LDH 692-1445 (U/L); total protein 5.7-8.1 (g/dL); albumin 2.1-3.6 (g/dL); globulin 3-4.9 (g/dL); BUN 6.0-27 (mg/dL); Creatinine 1.0-2.0 (mg/dL); triglyceride 0-14 (mg/dL); CPK 35-280 (U/L); P 5.6-6.5 (mg/dL); Mg 1.8-2.3 (mg/dL); Na 132-152 (mmol/L); K 3.9-5.8 (mmol/L); Cl 95-110 (mmol/L) [22].

Table S3. Effects of SeE or CZM supplementation on MLP, MUN, MSNFP, MTSP, MC and SCC data changes in transition dairy cattle.

Item[1]	Treatment[2]	Days after calving				
		7	14	21	30	60
**MLP (%)**	SeE	4.38^{b}	4.69^{b}	4.71^{b}	4.87^{b}	4.80^{b}
	CZM	4.65^{a}	4.95^{a}	4.97^{a}	5.03^{a}	5.03^{a}
	CON	4.40^{b}	4.76^{b}	4.83^{ab}	4.95^{ab}	4.82^{b}
	SEM	0.29	0.17	0.22	0.14	0.18
	treatment *p* value	0.01	< 0.001	< 0.01	< 0.01	< 0.001
	time *p* value	< 0.001				
	treat × time *p* value	0.40				
MUN (mg/dL)	SeE	14.5	14.3^{a}	13.6	15.0^{a}	14.2^{ab}
	CZM	11.3	11.5^{b}	12.6	13.3^{b}	15.5^{a}
	CON	14.3	11.9^{b}	12.3	13.5^{ab}	13.4^{b}
	SEM	3.92	2.35	1.95	1.97	2.18
	treatment *p* value	0.03	< 0.01	0.17	0.03	0.02
	time *p* value	< 0.001				
	treat × time *p* value	< 0.001				
MSNFP (%)	SeE	9.50	8.68^{ab}	8.37	8.38^{b}	8.37
	CZM	9.18	8.82^{a}	8.62	8.63^{a}	8.72
	CON	8.72	8.60^{b}	8.42	8.49^{ab}	8.37
	SEM	1.18	0.27	0.39	0.27	1.13
	treatment *p* value	0.17	0.05	0.15	0.03	0.62
	time *p* value	< 0.001				
	treat × time *p* value	0.38				
MTSP (%)	SeE	12.86	12.60	12.16	12.00	11.21
	CZM	13.32	12.53	12.53	12.38	12.58
	CON	13.52	12.80	12.43	12.12	11.95
	SEM	1.39	0.65	0.63	0.72	2.45
	treatment *p* value	0.39	0.46	0.13	0.30	0.29
	time *p* value	< 0.01				
	treat × time *p* value	0.89				
MC (mg/dL)	SeE	181.3	195.6^{a}	186.5	173.6	166.3
	CZM	188.8	176^{ab}	171.4	162.5	167.1
	CON	183.3	168.5^{b}	167.8	170.4	153.9
	SEM	31.02	31.59	25.60	23.43	19.34
	treatment *p* value	0.79	0.04	0.09	0.39	0.09
	time *p* value	< 0.001				
	treat × time *p* value	0.02				
SCC (10^3/ml)	SeE	875.0	229.3^{b}	247.0	132.9	436.5
	CZM	426.9	90.1^{b}	181.0	80.9	91.3
	CON	2612.0	1004.0^{a}	1001.0	481.6	601.7
	SEM	3337.6	758.5	1203.9	569.3	715.2
	treatment *p* value	0.15	< 0.001	0.10	0.09	0.12
	time *p* value	< 0.001				
	treat × time *p* value	0.14				

[1]MLP, milk lactose percentage; MUN, milk urea nitrogen, MSNFP, milk solids-not-fat percentage; MTSP, milk total solid percentage; MC, milk citrate; SCC, somatic cell counts.

[2]SeE: supplemented with 1,500 IU vitamin E and 0.3 ppm of organic Se, n = 8; CZM: supplemented with 12.5 ppm of organic Zn, 5 ppm of organic Cu and 7.5 ppm Mn, n = 8; CON: fed a basal diet without supplementation, n = 8. SEM, standard error of mean.

[ab], Values within a column with different superscripts differ treatment effect significantly ($p < 0.05$).
